# Importance of Core Genome Functions for an Extreme Antibiotic Resistance Trait

**DOI:** 10.1128/mBio.01655-17

**Published:** 2017-12-12

**Authors:** Larry A. Gallagher, Samuel A. Lee, Colin Manoil

**Affiliations:** Department of Genome Sciences, University of Washington, Seattle, Washington, USA; University of Chicago

**Keywords:** AB5075, Tn-seq, intrinsic resistance, mutant library, tobramycin

## Abstract

Extreme antibiotic resistance in bacteria is associated with the expression of powerful inactivating enzymes and other functions encoded in accessory genomic elements. The contribution of core genome processes to high-level resistance in such bacteria has been unclear. In the work reported here, we evaluated the relative importance of core and accessory functions for high-level resistance to the aminoglycoside tobramycin in the nosocomial pathogen *Acinetobacter baumannii*. Three lines of evidence establish the primacy of core functions in this resistance. First, in a genome scale mutant analysis using transposon sequencing and validation with 594 individual mutants, nearly all mutations reducing tobramycin resistance inactivated core genes, some with stronger phenotypes than those caused by the elimination of aminoglycoside-inactivating enzymes. Second, the core functions mediating resistance were nearly identical in the wild type and a deletion mutant lacking a genome resistance island that encodes the inactivating enzymes. Thus, most or all of the core resistance determinants important in the absence of the enzymes are also important in their presence. Third, reductions in tobramycin resistance caused by different core mutations were additive, and highly sensitive double and triple mutants (with 250-fold reductions in the MIC) that retained accessory resistance genes could be constructed. Core processes that contribute most strongly to intrinsic tobramycin resistance include phospholipid biosynthesis, phosphate regulation, and envelope homeostasis.

## IMPORTANCE

The inexorable increase in bacterial antibiotic resistance threatens to undermine many of the procedures that transformed medicine in the last century. One strategy to meet the challenge antibiotic resistance poses is the development of drugs that undermine resistance. To identify potential targets for such adjuvants, we identified the functions underlying resistance to an important class of antibiotics for one of the most highly resistant pathogens known.

## INTRODUCTION

The dramatic increase in the incidence of bacterial antibiotic resistance threatens health care in many ways (1). Six nosocomial pathogens that can exhibit high-level resistance to multiple antibiotics are of particular concern, one of which (*Acinetobacter baumannii*) is the subject of this study (2).

Extreme antibiotic resistance is generally associated with the expression of dedicated resistance functions encoded in accessory genomic elements. For example, *A. baumannii* strains often carry large genome islands with multiple resistance genes encoding antibiotic-inactivating enzymes, efflux pumps, and resistant target proteins (3–5).

Although accessory genes encode the most conspicuous determinants of antibiotic resistance, core genes alone can confer appreciable resistance (called intrinsic resistance) (6). Important intrinsic resistance functions limit outer membrane permeability, mediate broad-specificity drug efflux, and control stress tolerance (7–10). Exopolysaccharide production can also contribute to intrinsic resistance and may be induced by antibiotic exposure (11, 12). Additional intrinsic resistance functions that act by unknown mechanisms have been identified by unbiased mutant screening (6).

Do intrinsic resistance functions contribute significantly to the high-level resistance of bacteria expressing powerful accessory resistance functions? It could be that in highly resistant strains, the accessory functions would be sufficiently potent to supplant intrinsic resistance functions. Alternatively, high-level resistance might represent the sum of the contributions of both intrinsic and dedicated functions. Understanding which alternative is more accurate is critical because intrinsic resistance functions represent potential targets for broad-spectrum drugs to enhance antibiotic efficacy.

To evaluate the relative importance of accessory and intrinsic gene functions for a high-level resistance trait, we analyzed resistance to the aminoglycoside tobramycin in *A. baumannii* AB5075 (13). This strain is a recent clinical isolate resistant to tobramycin at ~100 times the Clinical and Laboratory Standards Institute breakpoint concentration. The strain carries a plasmid-borne resistance island that encodes five aminoglycoside-modifying enzymes, including two predicted to inactivate tobramycin (aminoglycoside 6′-*N*-acetyltransferase [AacA4] and 2′′-aminoglycoside nucleotidyltransferase [AadB]) (14).

In this study, we address two questions. First, to what extent do core functions contribute to high-level tobramycin resistance in a strain expressing multiple inactivating enzymes? To answer this question, we carried out saturation level identification of tobramycin resistance determinants in strain AB5075. Second, does the set of core resistance determinants differ in isogenic strains with and without accessory resistance genes? We addressed this question by comparing core resistance functions in the parent strain with those of a mutant with the principal genome resistance islands deleted. The results document the importance of a nearly identical set of core functions whether or not aminoglycoside-inactivating enzymes are expressed.

## RESULTS

### Tobramycin-sensitive mutants of *A. baumannii* AB5075.

**FIG 1:**
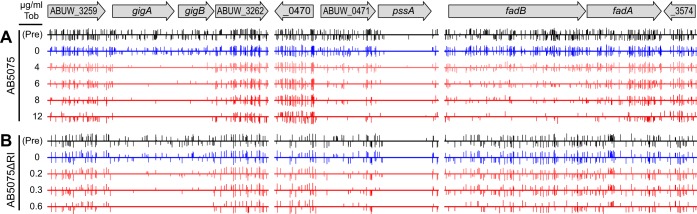
Mutations reducing tobramycin (Tob) resistance. Tn-seq read profiles for three regions of the genome with resistance genes are shown for the wild type (AB5075) (A) and the resistance island deletion strain (AB5075ΔRI) (B). Transposon insertion positions and orientations are represented by vertical bars with lengths corresponding to log_2_-transformed read counts. Growth in normally subinhibitory tobramycin (~10 to 40% of the MIC) leads to loss of transposon sequence reads for tobramycin resistance genes *gigA*, *gigB*, ABUW_0471, and *fadB* (in AB5075). Note the absence of insertions in *pssA* under all conditions because it is essential. The vertical scale corresponds to a maximum log_2_ (normalized number of reads per site) value of approximately 11. Pre, original mutant pool.

**FIG 2:**
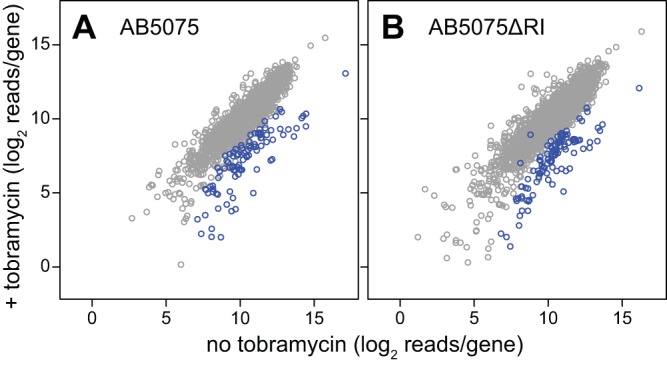
Genome scale identification of tobramycin resistance genes. Tn-seq read counts per gene after growth of mutant pools with and without tobramycin are shown for AB5075 (A) and AB5075ΔRI (B). Values represent the averages of all tobramycin levels analyzed for each strain. Genes whose mutants were significantly selected against during growth in the presence of tobramycin are represented by blue circles (see Materials and Methods). Individual plots of each tobramycin level examined are presented in Fig. S1. The exceptional blue circle near the *x*-*y* diagonal in panel B represents a gene whose mutants were significantly selected against at two of the three tobramycin levels analyzed.

We initially asked if there were functions other than the known modifying and efflux functions that contribute to the high-level tobramycin resistance of AB5075. To identify such functions at a comprehensive scale, we used transposon sequencing (Tn-seq) to screen for mutations that reduce tobramycin resistance. We grew a saturation level transposon mutant pool (~450,000 mutants) at several subinhibitory tobramycin levels and identified mutants that were lost (see Materials and Methods). The loss of insertions in several resistance genes after growth in the presence of tobramycin is illustrated in Fig. 1A. A plot comparing recovery with and without tobramycin for all of the genes represented in the mutant pool shows that insertions in numerous genes reduce resistance (Fig. 2A; and see Fig. S1 in the supplemental material). On the basis of a mutant fitness decrease based on statistical criteria described in Materials and Methods, the screening identified 105 top candidate resistance genes (Data Set S1). Although the group included the expected accessory tobramycin resistance genes, most (80%) of the genes were novel and belong to the core genome.

10.1128/mBio.01655-17.2 DATA SET S1  Candidate tobramycin resistance genes identified in AB5075 and AB5075ΔRI. Download DATA SET S1, XLSX file, 1.9 MB. Copyright © 2017 Gallagher et al. 2017 Gallagher et al. This content is distributed under the terms of the Creative Commons Attribution 4.0 International license.

10.1128/mBio.01655-17.4 FIG S1  Tn-seq identification of genes showing mutant sensitivity to tobramycin at specific concentrations. Plots of Tn-seq read counts per gene with and without tobramycin for each drug level analyzed are shown for AB5075 and AB5075ΔRI. Genes whose mutants were significantly negatively selected during growth in the presence of tobramycin at each level are represented by blue circles (see Materials and Methods). Note that the overall spread of data points increased with the drug concentration, presumably reflecting the greater stochastic loss of mutants at higher concentrations. The tobramycin MIC was 32 µg/ml for AB5075 and 2.0 µg/ml for AB5075ΔRI. Values plotted represent the log_2_-transformed normalized total number of reads per gene from all insertion locations between 5 and 90% of the gene’s coding sequence. Zero values are plotted adjacent to the axes. Essential genes are not shown. Download FIG S1, TIF file, 1.9 MB. Copyright © 2017 Gallagher et al. 2017 Gallagher et al. This content is distributed under the terms of the Creative Commons Attribution 4.0 International license.

Since Tn-seq assays monitor mutants grown in competition, modest reductions in the growth rate are amplified and it can be difficult to distinguish strong from weak phenotypes. Thus, it is critical to validate Tn-seq phenotypes with quantitative assays of individual mutants (15). Accordingly, we evaluated the tobramycin MICs for 437 individual mutants representing the genes of the top group and others with weaker Tn-seq phenotypes (195 genes total). We obtained mutants primarily from the arrayed AB5075 transposon mutant library (14), although we also created gene deletion mutants for five genes for which the phenotypes of different transposon mutants disagreed (see below). Altogether, we found that mutations in 32 genes strongly reduced resistance (≥4-fold MIC reductions) and mutations in another 49 genes led to weaker phenotypes (2-fold MIC decreases) (Table 1; Data Set S1).

**TABLE 1:** Tobramycin resistance genes^g^

Locus	Gene	Product	No. of Tn-seq identifications^a^		AB5075		AB5075ΔRI ΔMIC^c^^,^^d^
			AB5075 (of 4)	AB5075ΔRI (of 3)	No. of alleles^b^	ΔMIC^c^	
ABUW_0051		Histidine triad protein	0	1	1	4	ND^h^
ABUW_0091	*pgpA*	Phosphatidylglycerophosphatase A	4	1	5	8	8
ABUW_0097	*serB*	Phosphoserine phosphatase	4	2	2	16	32
ABUW_0257	*ompR*	Two-component response regulator	0	0	7(2Δ)	4	4
ABUW_0331	*serA*	d-3-Phosphoglycerate dehydrogenase	0	0	2	32	ND
ABUW_0355	*astE*	Succinylglutamate desuccinylase	1	0	2	16^e^	32
ABUW_0388	*corA*	Magnesium and cobalt transport protein	4	2	4	4	ND
ABUW_0460		Hypothetical protein	0	0	3	4	ND
ABUW_0471		SAM^j^-dependent methyltransferase	3	2	4	2–64^f^	2–64^f^
(ABUW_0472)	(*pssA*)	(Phosphatidylserine synthase)					
ABUW_0532	*yajC*	Preprotein translocase subunit	2	2	3	16^e^	8
ABUW_0622	*trpA*	Tryptophan synthase subunit	4	3	5	8	8
ABUW_0643	*cysI*	Sulfite reductase	1	3	4	4^*e*^	ND
ABUW_0644		Hypothetical protein	2	0	1	8	ND
ABUW_0699		Hypothetical protein	3	2	2	4	ND
ABUW_0924	*htpX*	Heat shock protein	4	3	6	4	4
ABUW_0949		Hypothetical protein	2	3	3	4	4
ABUW_1072	*idh*	Isocitrate dehydrogenase	3	2	2	8	ND
ABUW_1118	*pstB*	Phosphate ABC transporter subunit	3	1	2	8	8
ABUW_1242	*rlpA*	Rare lipoprotein A	0	0	2(1Δ)	8	8
ABUW_1451		GatB/YqeY domain protein	4	3	5(1Δ)	16^e^	8
ABUW_2104	*aspA*	Fumarate lyase	2	2	2	4	ND
ABUW_2299	*fxsA*	Suppressor of F exclusion of phage T7	4	3	2	4	ND
ABUW_2307	*bioB*	Biotin biosynthesis protein	3	3	2	4	4
ABUW_2357		Aspartate aminotransferase	0	3	3	4^*e*^	ND
ABUW_2865	*lon*	Lon protease	3	2	5	4^*e*^	ND
ABUW_2973	*mqo*	Malate dehydrogenase	0	0	1	4	ND
ABUW_3116		Pseudouridylate synthase	0	0	2	4^*e*^	ND
ABUW_3121	*bioH*	Biotin biosynthesis protein	4	2	1	4	2
ABUW_3260	*gigA*	Two-component response regulator	4	2	4(2Δ)	4^*e*^	8
ABUW_3261	*gigB*	Anti-anti-sigma factor	4	3	4(2Δ)	4^*e*^	8
ABUW_3360	*lptE*	LPS^k^ delivery protein	4	2	4	4	4
ABUW_3402	*tig*	Trigger factor	3	2	4	8^*e*^	4
ABUW_3572	*fadB*	Fatty acid oxidation complex subunit	4	1	2	4	4
ABUW_3573	*fadA*	Acetyl coenzyme A C-acyltransferase	1	1	5	4^*e*^	2
ABUW_3609		DNA-binding protein H-NS	0	0	2	8^*e*^	ND
ABUW_3638	*pbpG*	d-Ala–d-Ala carboxypeptidase	4	3	2	8	8
ABUW_4051	*aacA4*	Aminoglycoside 6′-*N*-acetyltransferase	4	NA^i^ (RI2)	5	16^e^	NA (RI2)
ABUW_4060	*aadB*	2′′-Aminoglycoside nucleotidyltransferase	3	NA (RI2)	4	8^*e*^	NA (RI2)
ABUW_5005		Hypothetical protein	4	3	1	4	ND

^a^ The number of tobramycin levels (of four for AB5075 and three for AB5075ΔRI) that displayed significant negative selection is shown (see Materials and Methods).

^b^ The number of unique mutant alleles tested in the AB5075 background. Parentheses indicate the number that were gene deletions [e.g., 5(1Δ) indicates that four transposon mutants and one deletion mutant were assayed in AB5075].

^c^ Under our assay conditions, the tobramycin MICs were 32 µg/ml for AB5075 and 2 µg/ml for AB5075ΔRI. By Clinical and Laboratory Standards Institute standard methods, the MICs were approximately 10-fold higher (unpublished data). ΔMIC, average fold decrease in the MIC of mutants relative to the parent strain. RI2, resistance island 2 gene.

^d^ For selected genes, one or two representative alleles were transferred from AB5057 into AB5075ΔRI and MICs were determined.

^e^ Greater-than-2-fold allelic variability was observed. Reported ΔMICs correspond to the phenotype of deletion alleles, when available, or the most common insertion phenotype.

^f^ ABUW_0471 mutations appear to be polar on ABUW_0472 (*pssA*) (see text).

^g^ Genes corresponding to mutants with tobramycin MIC reductions of at least 4-fold relative to the parent are listed. Consensus MICs from multiple mutant alleles are shown.

^h^ ND, not done.

^i^ NA, not applicable.

^j^ SAM, *S*-adenosylmethionine.

^k^ LPS, lipopolysaccharide.

### Other candidate resistance genes.

In addition to genes identified by Tn-seq, we also evaluated the mutant phenotypes of 33 genes potentially contributing to tobramycin resistance on the basis of annotated function. Most of the additional genes were predicted to encode efflux pumps (21 genes) or potential aminoglycoside-modifying functions (5 genes) or were orthologous to tobramycin resistance genes of *P. aeruginosa* (3 genes) (16–18) (Data Set S1). We assayed multiple insertion mutants for most of these genes, and for seven of them, we also created and assayed deletion mutants. Mutations inactivating 5 of the 33 genes reduced the tobramycin MIC ≥4-fold (Table 1; Data Set S1). The result shows that even extensive Tn-seq screening may fail to identify some mutants with significant phenotypes.

In total, we identified 37 genes that strongly contribute to tobramycin resistance in AB5075 (Table 1). All but three of these genes belong to the core genome, the exceptions being the two known tobramycin-modifying functions (*aadB* and *aacA4*) and a plasmid gene of unknown function (ABUW_5005). Mutations in six core genes (*serA*, *serB*, *astE*, ABUW_0471, *yajC*, and ABUW_1451) led to particularly strong phenotypes, individually increasing sensitivity as much as or more than the elimination of both tobramycin-modifying functions (see below). Overall, the results show the profound contribution of core genome functions to high-level aminoglycoside resistance, even when potent antibiotic-modifying enzymes are expressed.

The core genes we identified were also important for resistance to aminoglycosides other than tobramycin. We tested mutants corresponding to 20 of the genes for sensitivity to gentamicin and amikacin and found that nearly all exhibited reduced resistance to the two drugs, with MIC decreases ranging from 1.3- to 21-fold relative to a control insertion strain (Data Set S1).

### Allelic variability.

In the course of the single mutant studies, we identified several genes for which insertions at some positions strongly increased tobramycin sensitivity, while those at others did not (Data Set S1). Four such genes encode secreted or integral membrane proteins: three RND efflux pump membrane fusion proteins (ABUW_0034/*arpA*, ABUW_3153, and ABUW_3560) and a MATE family efflux pump (ABUW_3486/*abeM*). Since aberrant proteins targeted to the membrane can increase aminoglycoside sensitivity (18, 19), we suspected that gain-of-function effects could explain the allelic variability. Indeed, gene deletion mutants of each of the four genes displayed wild-type tobramycin sensitivity, suggesting that the insertion alleles that increased sensitivity did so by gain-of-function effects. However, we have not ruled out the alternative possibility that unlinked second-site mutations are responsible in some cases (Fig. S2).

10.1128/mBio.01655-17.5 FIG S2  Genes displaying allelic variability in tobramycin sensitivity. Insertion locations for T26 mutants in five AB5075 loci are shown as green triangles, with their positions above or below the line representing different transposon orientations, and the tobramycin MIC fold decreases are indicated. In all cases, a subset of alleles displayed significant tobramycin sensitivity (4- to 8-fold decreased MICs), while most alleles displayed no change in sensitivity. A mutant with an in-frame deletion of each gene (dotted lines) displayed wild-type tobramycin sensitivity, suggesting that the sensitive alleles probably represented gain-of-function mutations. Download FIG S2, EPS file, 0.7 MB. Copyright © 2017 Gallagher et al. 2017 Gallagher et al. This content is distributed under the terms of the Creative Commons Attribution 4.0 International license.

The plasmid-borne *aphA6* gene (ABUW_4087) also showed allelic variability (Fig. S2). This gene is predicted to encode a cytoplasmic aminoglycoside phosphotransferase that does not act on tobramycin (20). We found that a deletion eliminating *aphA6* and adjacent IS elements (21) did not change tobramycin sensitivity, again indicating that the insertions increasing sensitivity are gain-of-function alleles (Fig. S2).

### Tobramycin-sensitive mutants of a resistance island deletion strain.

**FIG 3:**
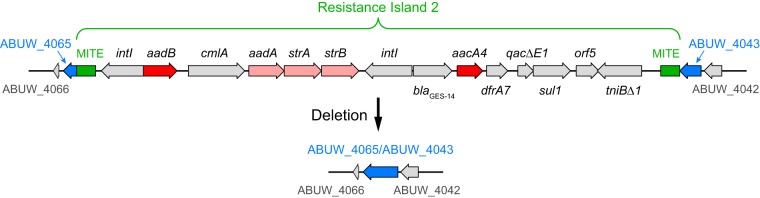
Deletion of resistance island 2. Resistance island 2 (14) resides on plasmid p1 and contains two genes for modification enzymes predicted to inactivate tobramycin, *aadB* and *aacA4* (red), as well as genes for three other aminoglycoside modification functions (pink). The precise deletion indicated restores the ancestral gene of unknown function (ABUW_4065/ABUW_4043, blue) interrupted by the island (Text S1). Genetic elements: MITE, miniature inverted-repeat transposable element; *intI*, integrase; *aadB*, aminoglycoside 2′′-nucleotidyltransferase; *cmlA*, chloramphenicol resistance protein; *aadA*, aminoglycoside 3′′-nucleotidyltransferase; *strA* and *strB*, streptomycin phosphotransferases; *bla*_GES-14_, class A β-lactamase; *aacA4*, aminoglycoside 6′-acetyltransferase; *dfrA7*, dihydrofolate reductase; *qacE*Δ*1*, multidrug resistance efflux pump; *sul1*, sulfonamide resistance protein; *orf5*, unknown function; *tniB*Δ*1*, transposition protein.

We next evaluated whether there are intrinsic resistance determinants that function in the absence of tobramycin-modifying functions but not in their presence. To do this, we first created an AB5075 derivative lacking the strain’s two major accessory genome resistance islands (14), including genes for the two tobramycin-modifying enzymes (*aacA4* and *aadB*) (Fig. 3). As expected, the strain (AB5075ΔRI) was much more tobramycin sensitive than its parent, with a 16-fold reduction in the MIC (Table 2).

**TABLE 2:** Tobramycin sensitivities of multiple core gene mutants

Genotype^a^	ΔMIC^b^
Wild type (AB5075)	1
ΔRI1 ΔRI2 (AB5075ΔRI)	16
	
Single mutants	
ABUW_0471::T26	64
*astE*::T26	16
*serB*::T26	16
*pstB*::T26	8
*htpX*::T26	4
ΔABUW_3260-1	4
	
Double mutants	
*serB*::T26rec ABUW_0471::T26	256
*astE*::T26rec *serB*::T26	32
*pstB*::T26rec *serB*::T26	32
*htpX*::T26rec *astE*::T26	32
*htpX*::T26rec *pstB*::T26	16
	
Triple mutants	
*htpX*::T26rec *astE*::T26rec ABUW_0471::T26	128
*htpX*::T26rec *pstB*::T26rec ABUW_0471::T26	128
*pstB*::T26rec *serB*::T26rec ΔABUW_3260-1	64
*htpX*::T26rec *pstB*::T26rec ΔABUW_3260-1	16

^a^ T26rec, insertion after removal of tetracycline resistance marker from T26 (see Materials and Methods).

^b^ ΔMIC, fold decrease in the MIC for the mutant relative to that for AB5075.

**FIG 4:**
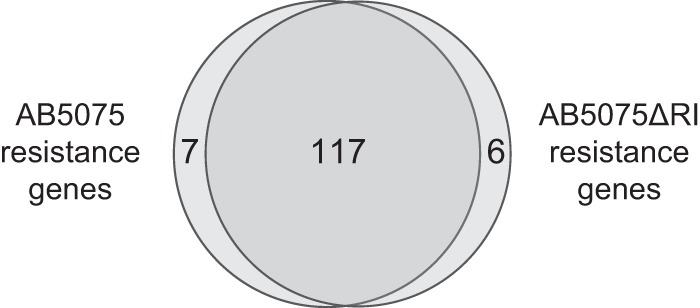
Shared core resistance genes in the wild-type and resistance island deletion strains. The overlap of the top resistance genes identified by Tn-seq in AB5075 (105 genes) and AB5075ΔRI (108 genes) is shown. The 130 unique genes represented do not include 4 resistance island genes and 1 resistance gene essential in AB5075ΔRI. A gene was included in the overlap region if (i) it was identified independently in both strains or (ii) the ratio of Tn-seq read counts in the presence of tobramycin for the two strains did not differ significantly from that of the preponderance of AB5075 genes (Fig. S3).

We next employed Tn-seq analysis to identify tobramycin resistance determinants in the deletion mutant background by using an approach analogous to that used with the wild type (Fig. 1B and 2B). The screening identified 108 genes whose mutants were strongly depleted during growth with tobramycin (see Materials and Methods and Data Set S1). There was a striking overlap of these resistance genes with those identified in the parent strain (Fig. 4; and see Fig. S3). The findings imply that nearly identical intrinsic resistance functions are important whether or not the tobramycin-modifying functions are present.

10.1128/mBio.01655-17.6 FIG S3  Similarity of mutant depletion of core resistance functions in AB5075 and AB5075ΔRI during growth with tobramycin. The distributions of log_2_-transformed read ratios for the top 130 tobramycin resistance genes identified by Tn-seq analysis (see Fig. 4) in either AB5075 or AB5075ΔRI (histogram bars, scales to the left) and for all other nonessential genes (distribution lines, scales to the right) are shown for the following comparisons: AB5075 grown with and without tobramycin (A), AB5075ΔRI grown with and without tobramycin (B), and AB5075 and AB5075ΔRI grown with tobramycin (C). When the ratios for all nonessential genes in panel C were modeled as a normal distribution, 23 of the 130 resistance genes fell significantly outside the distribution (*P* < 0.05 or *P* > 0.95), 13 of which had been identified in only one of the two parent strains by Tn-seq (Fig. 4). The read ratios were calculated by using the average normalized number of reads per gene from all tobramycin levels analyzed per strain. WT, AB5075; ΔRI, AB5075ΔRI. Download FIG S3, EPS file, 0.9 MB. Copyright © 2017 Gallagher et al. 2017 Gallagher et al. This content is distributed under the terms of the Creative Commons Attribution 4.0 International license.

Of the genes that appeared to be necessary for resistance in one strain but not the other (on the basis of the Tn-seq results), the greatest difference was seen in *fadB* (Fig. 1, right panel). However, when we transferred *fadB* (and *fadA*) insertion alleles from AB5075 into AB5075ΔRI, we found that the mutations led to the same MIC decrease (4-fold) in both strain backgrounds (Table 1). Although the basis of the difference in the behavior of the mutants in Tn-seq pools and as individual strains is not clear, the result shows that the contributions of *fadAB* to resistance do not depend on the tobramycin-modifying functions.

While carrying out these screenings, we were surprised to find four genes that were essential for the growth of AB5075ΔRI, but not that of AB5075, on all of the growth media used. These were genes for dihydrofolate reductase (*folA*), dihydropteroate synthase (*folP*), thioredoxin (*trxB*), and glutathione *S*-transferase (*gst*/ABUW_2725). The first three of these genes are paralogs of genes in the resistance islands, suggesting that the resistance island genes can function in place of the corresponding core genes for normal growth. The fourth gene (*gst*) lacks paralogs in the resistance islands, and the basis of its essentiality in AB5075ΔRI is unclear.

In addition, by phenotype microarray analysis (22), we found that AB5075ΔRI had become sensitive to two boron-containing compounds, boric acid and sodium metaborate. A single resistance island 1 (RI1) deletion mutant was also sensitive, and single transposon mutant analysis of seven genes within RI1 identified *sup* (ABUW_3659) (annotated as encoding a sulfate permease) as the gene responsible (data not shown).

### Single mutant phenotypes in AB5075ΔRI.

**FIG 5:**
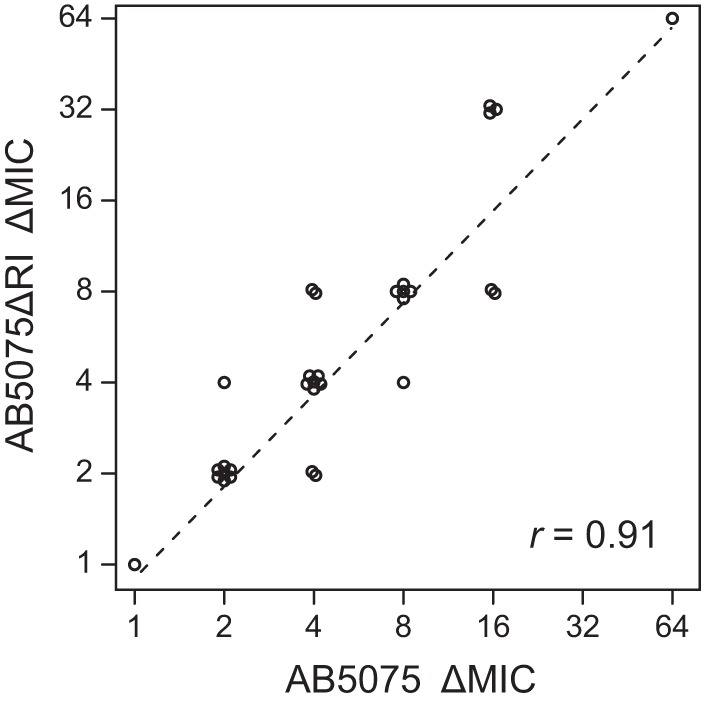
Direct comparison of resistance mutation effects in the presence and absence of tobramycin modification functions. The tobramycin MIC decreases (ΔMICs) caused by identical insertion or deletion mutations affecting 24 resistance genes were compared in the AB5075 and AB5075ΔRI genetic backgrounds (Pearson’s *r* = 0.91, *n* = 31, *P* = 2.5 × 10^−12^). A least-squares regression line of the log_2_-transformed ΔMICs is fitted (dashed line).

To provide a direct comparison of core sensitizing mutations in the wild-type and resistance island-lacking strains, we transformed insertion alleles from the wild type into AB5075ΔRI or constructed identical deletion mutations in the two strains. Thirty-one such mutations (corresponding to 24 genes) were examined, and all reduced tobramycin sensitivity comparably in the two strain backgrounds (Fig. 5). The results thus imply that individual core function contributions to resistance are quantitatively similar whether or not the tobramycin-modifying enzymes are expressed.

### Importance of phospholipid biosynthesis in intrinsic tobramycin resistance.

Three genes with strong mutant phenotypes appear to affect phospholipid synthesis. The first of these encodes a phosphatase (PgpA) that catalyzes the final step in phosphatidylglycerol (PG) synthesis. Although some PG synthesis is probably essential (and indeed, an enzyme acting immediately prior to PgpA [PgsA] is essential) (14), we assume that other phosphatases can partially substitute for a missing PgpA (23). However, the *pgpA* mutation presumably alters membrane phospholipid makeup (or leads to precursor accumulation) sufficiently to increase tobramycin sensitivity.

A second set of mutations appears to affect PssA, the enzyme responsible for the synthesis of phosphatidylserine (PS) from CDP-diacylglycerol and serine. Although mutations in *pssA* are absent from the Tn-seq mutant pools because the gene is essential, insertions in the gene transcriptionally upstream of it (ABUW_0471) markedly reduced tobramycin resistance (MIC decreases of up to 64-fold). The reduction appears to be due to a polar effect on *pssA* on the basis of two observations. First the ABUW_0471 insertion alleles were phenotypically variable, with insertions closer to the 5′ end of the gene stronger than those closer to the 3′ end, a hallmark of nonsense-mediated polarity (24). A similar pattern is evident in the Tn-seq data (Fig. 1, middle panel). Second, an in-frame ABUW_0471 deletion mutation designed to be nonpolar did not reduce tobramycin resistance. We thus assume that decreased expression of *pssA* in the ABUW_0471 insertion mutants limits the production of PS (and its decarboxylation product phosphatidylethanolamine) and that the defect increases tobramycin sensitivity.

Finally, mutations in a serine biosynthesis gene (*serB*) led to enhanced tobramycin sensitivity, again presumably because of reduced PS synthesis. The mutants grew slowly on unsupplemented nutrient growth medium (LB), suggesting that the level of serine in the medium is not sufficient to fully supplement their auxotrophies. Two other serine pathway genes were either fully (*serC*) or borderline (*serA*) essential (14) (Fig. S4), presumably because of reduced phospholipid synthesis. Two very slowly growing *serA* mutants available in the arrayed AB5075 transposon mutant library displayed 32-fold decreases in the tobramycin MIC relative to that of the parent strain, further documenting the need for serine synthesis for intrinsic resistance.

10.1128/mBio.01655-17.7 FIG S4  Tn-seq data for genes encoding the serine biosynthesis pathway. The genomic regions that include *serA*, *serB*, and *serC* are shown in the format of Fig. 1. *serA* mutants appear to grow slowly and are lost during growth with or without tobramycin (except for some insertions near the 3′ end of the gene). *serB* mutants grow in the absence of the drug but are lost in the presence of tobramycin. The *serC* gene appears to be essential, and *serC* mutants are absent from the starting mutant pool. Download FIG S4, EPS file, 0.9 MB. Copyright © 2017 Gallagher et al. 2017 Gallagher et al. This content is distributed under the terms of the Creative Commons Attribution 4.0 International license.

Other than ABUW_0471 (see above), 24 of the genes in Table 1 reside in potential operons in which polar effects on genes downstream are possible. In nearly all of these cases, downstream genes were not essential and insertion mutants of them did not show altered tobramycin sensitivity on the basis of Tn-seq, suggesting that polar effects were not responsible for the sensitivity phenotypes (not shown). However, in four cases, immediate downstream genes were essential, and we cannot rule out the possibility that polar effects contribute to the observed sensitivity phenotypes. The four genes were *pgpA* (upstream of *glmU* and *glmS*), *yajC* (upstream of *secD* and *secF*), *trpA* (upstream of *accD*, *folC*, and ABUW_0625), and *lptE* (upstream of *holA*).

### Highly sensitized strains lacking multiple core resistance functions.

We sought to determine whether it is possible to create highly tobramycin-sensitive AB5075 strains by combining multiple mutations compromising core resistance. Indeed, several double and triple mutants had as much as 256-fold increased tobramycin sensitivity despite expressing the tobramycin-modifying functions (Table 2).

### Mutations increasing tobramycin resistance.

Our screenings identified several genes in which insertion mutations increased resistance to tobramycin (Data Set S1). Two of the genes encode a two-component regulator (PhoBR) controlling the bacterial response to inorganic phosphate limitation (25). Assays of six individual transposon mutants from the arrayed mutant library found that insertions in each gene increased the tobramycin MIC 4-fold (Data Set S1). This result implies that PhoBR activity reduces the tobramycin resistance of the wild type. To test explicitly whether PhoBR activation by phosphate limitation reduces resistance, we assayed the tobramycin MICs for strains grown on a defined medium supplemented with different levels of phosphate (Table 3). We found that wild-type strains were indeed sensitized by phosphate limitation, whereas *phoR* mutants were not. This finding confirms that activation of the PhoBR regulator by phosphate limitation enhances tobramycin sensitivity in AB5075. The finding also suggests that the increased tobramycin sensitivity of Pst phosphate transporter mutants (Table 1) could be explained by their activation of PhoBR (25).

**TABLE 3:** Limiting phosphate increases tobramycin sensitivity of AB5075

Strain	Genotype	MIC^a^ with PO_4_ at:	
		2 mM	10 µM
AB5075	Wild type	8	4
MAB02912	ABUW_1075::T26 (“wild-type” insertion control)	4	2
MAB00292	*phoR*::T26 (allele 1)	8	8
MAB21488	*phoR*::T26 (allele 2)	8	8

^a^ MICs were determined with duplicate efficiency-of-plating assays.

Mutations that impair electron transport can result in enhanced aminoglycoside resistance in other bacteria (26), but we did not identify such mutations in our screenings. However, in AB5075, many of the genes encoding central electron transport functions (including *nuoA* to *nuoN* [NADH dehydrogenase], *cyoA* to *cyoE* [cytochrome oxidase], and ubiquinone biosynthesis genes) were essential under the growth conditions used in our screenings. Thus, the effects of corresponding mutations on tobramycin resistance could not be evaluated.

## DISCUSSION

Our results show the importance of core gene function in high-level aminoglycoside resistance in *A. baumannii*. Three lines of evidence support this conclusion. First, in comprehensive screenings, core gene mutations accounted for 80% of the mutations sensitizing cells to tobramycin. Several core gene mutations led to phenotypes stronger than those caused by the elimination of two dedicated tobramycin-inactivating enzymes encoded in an accessory genome resistance island. Second, the effect of core gene functions on resistance were nearly identical in the wild type and a deletion mutant lacking the resistance island, indicating that the same mechanisms underlie intrinsic resistance in the two strains. Third, strains carrying multiple core gene mutations exhibited up to 250-fold tobramycin MIC decreases, despite the expression of the inactivating enzymes. Thus, strains expressing potent tobramycin-inactivating functions can be highly sensitive to the antibiotic if resistance functions encoded in the core genome are compromised.

The results imply that core and accessory tobramycin resistance functions act independently of one another, with high-level resistance reflecting the combined contributions of the two classes of determinants. The findings indicate that aminoglycosides kill bacteria by the same mechanisms at the elevated levels needed to inhibit the growth of highly resistant bacteria as at the lower levels needed for more sensitive strains. It remains to be determined whether high-level resistance to other antibiotics associated with potent accessory inactivating enzymes, such as β-lactams, also depends strongly on core gene function.

We found that validation of Tn-seq results with individual mutants was critical to defining the strengths of sensitivity phenotypes. Relatively extensive verification was feasible because of the availability of an arrayed single-mutant library with multiple alleles for most AB5075 genes.

Aminoglycosides are thought to kill bacteria by inducing translational errors that result in the production of membrane-destabilizing polypeptides (27). This mechanism could explain how several core gene mutations we identified strongly sensitized cells to tobramycin. For example, mutations that interfere with phospholipid biosynthesis (*pgp*, *pssA*, and *serB*) may destabilize the cytoplasmic membrane and make it more vulnerable to mistranslated polypeptides. The mutations could also increase permeability to the antibiotic. Likewise, mutations in the *gigAB* stress response genes could contribute to resistance by helping bacteria tolerate mistranslation products (28, 29). We were surprised to identify mutations affecting the tricarboxylic acid cycle that reduced tobramycin resistance (*idh*, *aspA*, and *mqo*), since efficient energy metabolism (proton motive force generation) is thought to promote aminoglycoside uptake (27). We suspect that these mutations have pleiotropic effects that account for the increases in sensitivity.

Orthologues of a number of the *A. baumannii* intrinsic tobramycin resistance genes have also been found in screenings for aminoglycoside-sensitive mutants of *Pseudomonas aeruginosa*, including *pgpA*, *gigAB*, *pstABC* (inorganic phosphate transport), *adeABC* (efflux), *fadAB* (fatty acid oxidation), *tig* (trigger factor), and *edd* (central carbon metabolism) mutants (16–18, 30). The importance of the corresponding intrinsic resistance mechanisms thus extends to species other than *A. baumannii*.

Molecules inhibiting intrinsic resistance functions represent potential adjuvants that can be used to enhance the efficacy of established antibiotics (31). Our finding that the same functions are active in *A. baumannii* strains exhibiting dramatically different levels of resistance indicates that targeting them could be effective against a wide range of isolates.

## MATERIALS AND METHODS

### Media.

The growth medium used was LB (10 g of tryptone, 5 g of yeast extract, and 8 g of NaCl per liter) solidified in LB agar with 15 g/liter agar. For phosphate analysis, we used morpholinepropanesulfonic acid (MOPS) minimal medium (32) with 15 mM sodium succinate and solidified with agarose.

### Strains.

Strain AB5075 (MAB101) and its arrayed mutant library carrying insertions of tetracycline resistance transposon T26 have been described previously (13, 14). AB5075ΔRI (MAB104) carries precise deletions of the two AB5075 resistance islands (14, 33) and was constructed by a suicide plasmid integration-excision method (Text S1). Targeted deletions of individual genes were created either by natural transformation of marked linear replacement constructs (29) or by the suicide plasmid methodology (Text S1 and Tables S1 and S2). Double mutants were created from strains in the transposon mutant library by excising the T26 tetracycline resistance determinant from a mutant, leaving an unmarked insertion, and then transforming genomic DNA from a second mutant, selecting for tetracycline-resistant recombinants. Triple mutants were made by repeating the process (Text S1). Nearly all (>90%) of the strains analyzed in these studies were opaque phase variants (34) (Text S1). For a complete list of the strains used in this study, see Data Set S2.

10.1128/mBio.01655-17.8 TABLE S1  Deletion strain construction summary information. Download TABLE S1, DOCX file, 0.01 MB. Copyright © 2017 Gallagher et al. 2017 Gallagher et al. This content is distributed under the terms of the Creative Commons Attribution 4.0 International license.

10.1128/mBio.01655-17.9 TABLE S2  Sequences of the oligonucleotide primers used in this study. Download TABLE S2, DOCX file, 0.01 MB. Copyright © 2017 Gallagher et al. 2017 Gallagher et al. This content is distributed under the terms of the Creative Commons Attribution 4.0 International license.

10.1128/mBio.01655-17.3 DATA SET S2  Strains used in this study. Download DATA SET S2, XLSX file, 0.1 MB. Copyright © 2017 Gallagher et al. 2017 Gallagher et al. This content is distributed under the terms of the Creative Commons Attribution 4.0 International license.

10.1128/mBio.01655-17.1 TEXT S1  Supplemental materials and methods used in this study. Download TEXT S1, DOCX file, 0.02 MB. Copyright © 2017 Gallagher et al. 2017 Gallagher et al. This content is distributed under the terms of the Creative Commons Attribution 4.0 International license.

### Transposon mutant pools and tobramycin treatment.

The transposon mutant pools of AB5075 and AB5057ΔRI were created by transformation of transposon-transposase complexes of tetracycline resistance transposon T26 and consist of ~450,000 and ~120,000 insertion mutants, respectively (14). For Tn-seq identification of tobramycin-sensitive strains, mutant pools were grown on LB agar in the presence of different subinhibitory levels of tobramycin (Text S1). After 10 doublings, cells were harvested and genomic DNA was prepared with a Qiagen Tissue kit (Qiagen, Inc.).

### Tn-seq molecular processing and data analysis.

Genomic DNA from the mutant pools was processed by either the circle or the TdT method (14, 35). Custom scripts were used to map and count sequence reads. Read counts were normalized as described in Text S1. Normalized read counts for all genes are listed in Data Set S1. Gene level fitness difference for growth with and without tobramycin was the primary criterion used to define genes showing selection at each tobramycin level (36) (Text S1). The set of negatively selected genes was refined by using Wilcoxon rank sum tests to eliminate genes with low read counts and/or represented by insertions with variable behavior (Text S1).

### Tobramycin sensitivity assays.

MIC assays evaluated the growth of spots of bacteria (containing between 5 × 10^3^ and 5 × 10^5^ viable cells) and by efficiency of colony formation on LB agar containing tobramycin (Text S1).

### Accession number(s).

The sequence reads obtained in this study have been deposited in the Sequence Read Archive (https://www.ncbi.nlm.nih.gov/sra) under the accession numbers shown in Text S1 and Table S3.

10.1128/mBio.01655-17.10 TABLE S3  Tn-seq run summary information. Download TABLE S3, DOCX file, 0.02 MB. Copyright © 2017 Gallagher et al. 2017 Gallagher et al. This content is distributed under the terms of the Creative Commons Attribution 4.0 International license.
