# Supplementary figures and images for: Importance of Core Genome Functions for an Extreme Antibiotic Resistance Trait
Source: mBio. 2017 Dec 12;8(6):e01655-17. doi: 10.1128/mBio.01655-17 (PMC5727411; doi:10.1128/mBio.01655-17)

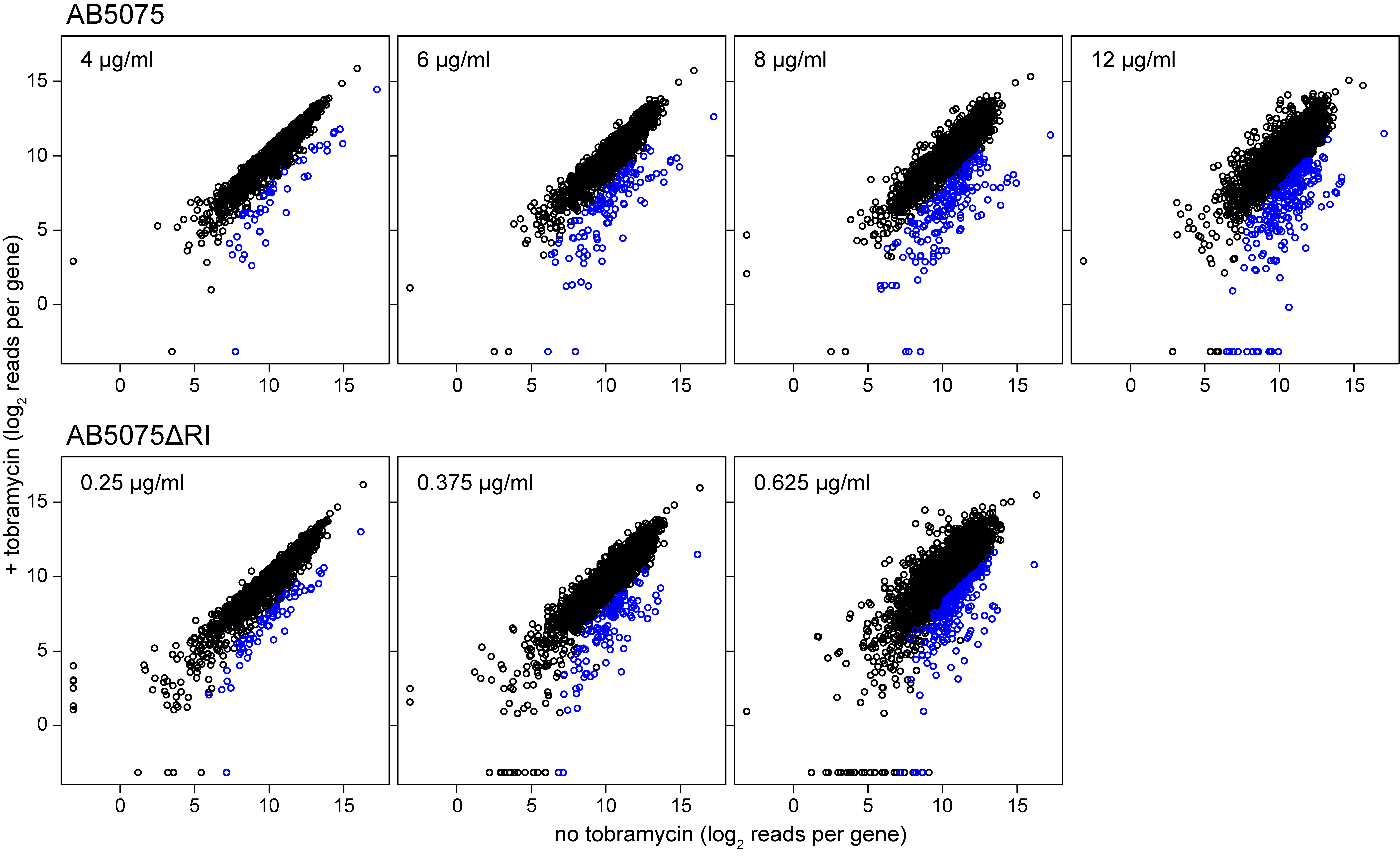

Supplement: FIG S1 [file mbo006173636sf1.tif]
