# Supplementary material for: Importance of Core Genome Functions for an Extreme Antibiotic Resistance Trait
Source: mBio. 2017 Dec 12;8(6):e01655-17. doi: 10.1128/mBio.01655-17 (PMC5727411; doi:10.1128/mBio.01655-17)
Supplement: TABLE S1 [file mbo006173636st1.docx]

**Table S1. Deletion strain construction summary information.**

| Strain | Targeted Locus | Gene(s) | Method* | 5’ flank primers† | 3’ flank primers† | Overlap extension primers† |
| --- | --- | --- | --- | --- | --- | --- |
| MAB105 | ABUW_0034 | *arpA* | Nat.Tfm. | 0034upF2, 0034upR2+ | 0034dnF2+, 0034dnR2 | 0034upF2,  0034dnR2 |
| MAB106 | ABUW_0256 | *envZ* | Nat.Tfm. | 0256upF2, 0256upR2+ | 0256dnF2+, 0256dnR2 | 0256upF2,  0256dnR2 |
| MAB107 | ABUW_0257 | *ompR* | Nat.Tfm. | 0257upF2, 0257upR2+ | 0257dnF2+, 0257dnR2 | 0257upF2,  0257dnR2 |
| MAB108 | ABUW_0257-6 | *envZ*, *ompR* | Nat.Tfm. | 0257upF2, 0257upR2+ | 0256dnF2+, 0256dnR2 | 0257upF2,  0256dnR2 |
| MAB109 | ABUW_0471 | *-* | Int.Exc. | 0471_5’F.1, 0471_5’R.1 | 0471_3’F.1, 0471_3’R.1 | 0471_5’F+Pst.1, 0471_3’R+Sac.1 |
| MAB110 | ABUW_0844-2 | *adeIJK* | Int.Exc. | 0844_5’F.1, 0844_5’R.2(42) | 0842_3’F.2, 0842_3’R.2 | 0844_5’F+Pst.1, 0842_3’R+Sac.2 |
| MAB111 | ABUW_0844-3 | *adeIJ* | Int.Exc. | 0844_5’F.1, 0844_5’R.1(43) | 0843_3’F.1, 0843_3’R.1 | 0844_5’F+Pst.1, 0843_3’R+Sac.1 |
| MAB112 | ABUW_1242 | *rlpA* | Nat.Tfm. | 1242upF2, 1242upR2+ | 1242dnF2+, 1242dnR2 | 1242upF2,  1242dnR2 |
| MAB113 | ABUW_1451 | - | Nat.Tfm. | 1451upF2, 1451upR2+ | 1451dnF2+, 1451dnR2 | 1451upF2,  1451dnR2 |
| MAB114 | ABUW_1974-5 | *adeAB* | Int.Exc. | 1974_5’F.1, 1974_5’R.1(75) | 1975_3’F.1, 1975_3’R.1 | 1974_5’F+Pst.1, 1975_3’R+Sac.1 |
| MAB115 | ABUW_1974-6 | *adeABC* | Int.Exc. | 1974_5’F.1, 1974_5’R.2(76) | 1976_3’F.2, 1976_3’R.2 | 1974_5’F+Pst.1, 1976_3’R+Sac.2 |
| MAB116 | ABUW_3153 | - | Nat.Tfm. | 3153upF2, 3153upR2+ | 3153dnF2+, 3153dnR2 | 3153upF2,  3153dnR2 |
| MAB117 | ABUW_3260 | *gigA* | Nat.Tfm. | 3260upF2, 3260upR2+ | 3260dnF2+, 3260dnR2 | 3260upF2,  3260dnR2 |
| MAB118 | ABUW_3260-1 | *gigA*, *gigB* | Nat.Tfm. | 3260upF2, 3260upR2+ | 3261dnF3+, 3261dnR3 | 3260upF2,  3261dnR3 |
| MAB119 | ABUW_3261 | *gigB* | Nat.Tfm. | 3261upF2, 3261upR2+ | 3261dnF3+, 3261dnR3 | 3261upF2,  3261dnR3 |
| MAB120 | ABUW_3486 | *abeM* | Nat.Tfm. | 3486upF2, 3486upR2+ | 3486dnF2+, 3486dnR2 | 3486upF2,  3486dnR2 |
| MAB121 | ABUW_3560 | *-* | Nat.Tfm. | 3560upF2, 3560upR2+ | 3560dnF2+, 3560dnR2 | 3560upF2,  3560dnR2 |
| MAB122 | ABUW_4087,  flanks | Tn*aphA6* | Int.Exc. | 4087_5’Fwd_1, 4087_5’Rev_1 | 4087_3’Fwd_1, 4087_3’Rev_1 | 4087_5’Fwd+Pst_1, 4087_3’Rev+Sac_1 |
| AB5075 ΔRI2 | RI2 | (island) | Int.Exc. | RI2_5'_FW3,  RI2_5'_RV3_Hom | RI2_3'_FW7,  RI2_3'_RV7 | RI2_5'_FW3+Res,  RI2_3'_RV7+Res |
| AB5075 ΔRI1† | RI1 (pΔRI1H)‡ | (island) | Int.Exc. | RI1_5'_FW6,  RI1_5'_RV6_Hom | RI1_3'_FW6_Hom,  RI1_3'_RV6 | RI1_5'_FW6+Hind,  RI1_3'_RV6+Sac |
| AB5075 ΔRI1† | RI1 (pΔRI1B)‡ | (island) | Int.Exc. | RI1_5'_FW6,  RI1_5'_RV6_Hom | RI1_3'_FW6_Hom,  RI1_3'_RV6 | RI1_5'_FW6+Bam,  RI1_3'_RV6+Sac |

* Nat.Tfm., natural transformation of marked linear replacement construct; Int.Exc., suicide plasmid integration-excision method (Methods and Text S1).

† Primer sequences are reported in Table S4.

‡ plasmid pΔRI1H was modified by deletion before conjugation. The resulting deletion strain contained a modified allele of *comM* (Methods). Subsequently, pΔRI1B was used to restore the wild-type *comM* gene.
