# Supplementary material for: Importance of Core Genome Functions for an Extreme Antibiotic Resistance Trait
Source: mBio. 2017 Dec 12;8(6):e01655-17. doi: 10.1128/mBio.01655-17 (PMC5727411; doi:10.1128/mBio.01655-17)
Supplement: TABLE S2 [file mbo006173636st2.docx]

**Table S2. Oligonucleotide primer sequences.**

| Primer | Sequence |
| --- | --- |
| T26xLoxF2 | ATCGATGAATTTTCTCGGGTGT |
| T26xLoxR2 | TGAAGCTCTTGTTGGCTAGTGC |
| 0034upF2 | TGGCAAAATTGAGGAAGTGAGA |
| 0034upR2+ | AACACCCGAGAAAATTCATCGATGTGACCATGCAAACAATCATC |
| 0034dnF2+ | CGCACTAGCCAACAAGAGCTTCATTGATCGTGAAAATCGTGCAG |
| 0034dnR2 | ATGCCAGCAAAAGTCATTGCTA |
| 0256upF2 | ATACTGAGGGCTTGTTGGCAAT |
| 0256upR2+ | AACACCCGAGAAAATTCATCGATGCCGCAGAACGTGGCTTAAT |
| 0256dnF2+ | CGCACTAGCCAACAAGAGCTTCAGTGAACAAGGCGGTTTAGAGGT |
| 0256dnR2 | CGACCTTCAGCCACTTCTTTTT |
| 0257upF2 | CTTTCGCCTTGAAAGATTTGCT |
| 0257upR2+ | AACACCCGAGAAAATTCATCGATTCGTCATCGACCACTAAAATGC |
| 0257dnF2+ | CGCACTAGCCAACAAGAGCTTCATACGCCGCTTAATTGAAGATAACC |
| 0257dnR2 | GGGGTTTAAACTGAACCACCAA |
| 0471_5'F.1 | TTTTGTGAGCCAATGACGAG |
| 0471_5'R.1 | GATCTGCTTGTTCGGAGAGGATCCAACGGTTGAGAAGCTGTAGCA |
| 0471_3'F.1 | CTACAGCTTCTCAACCGTTGGATCCTCTCCGAACAAGCAGATCAA |
| 0471_3'R.1 | AAGTGGTGCAGGCAGGTTAG |
| 0471_5'F+Pst.1 | GGCGCTGCAGTTTTGTGAGCCAATGACGAG |
| 0471_3'R+Sac.1 | GGCGGAGCTCAAGTGGTGCAGGCAGGTTAG |
| 0844_5'F.1 | CGTCAGCGAACTTCATTCAA |
| 0844_5'R.2(42) | CTTTTAAACCGCCACCTAGGATCCTAGCCGACATCATTGTTCCA |
| 0842_3'F.2 | GAACAATGATGTCGGCTAGGATCCTAGGTGGCGGTTTAAAAGCA |
| 0842_3'R.2 | CAGCCGAACTCCCATAATGT |
| 0844_5'F+Pst.1 | GGCGCTGCAGCGTCAGCGAACTTCATTCAA |
| 0842_3'R+Sac.2 | GGCGGAGCTCCAGCCGAACTCCCATAATGT |
| 0844_5'R.1(43) | GCATCACGATTTATGCTCCTGGATCCTAGCCGACATCATTGTTCCA |
| 0843_3'F.1 | GAACAATGATGTCGGCTAGGATCCAGGAGCATAAATCGTGATGCAA |
| 0843_3'R.1 | TGAGCAATGCGTTTTACAGG |
| 0843_3'R+Sac.1 | GGCGGAGCTCTGAGCAATGCGTTTTACAGG |
| 1242upF2 | AAAACCAATATGCCGATGCTCT |
| 1242upR2+ | AACACCCGAGAAAATTCATCGATATGCTTAAACTAGCCGTCAGTGC |
| 1242dnF2+ | CGCACTAGCCAACAAGAGCTTCAGTGCAGCTAAACGCCTTGGTAT |
| 1242dnR2 | AGACCGGACCAATCAAGAGAAA |
| 1451upF2 | GACTTTGTCGAAAGCCTCACCT |
| 1451upR2+ | AACACCCGAGAAAATTCATCGATTCGATTTGCTTAATTGCTGCTTG |
| 1451dnF2+ | CGCACTAGCCAACAAGAGCTTCAATTCTCTACGTCCGATCATAGCC |
| 1451dnR2 | ATGTTGGGTACACAAGCAGCAC |
| 1974_5'F.1 | TGCATCGCTATCATTCATGC |
| 1974_5'R.1(75) | TTTTCTACTGCACCCAAAAGGATCCCCTGTAATATCAGCCCAAT |
| 1975_3'F.1 | TGGGCTGATATTACAGGGGATCCTTTTGGGTGCAGTAGAAAAGC |
| 1975_3'R.1 | ATTTTGCGGAACAGGATGAC |
| 1974_5'F+Pst.1 | GGCGCTGCAGTGCATCGCTATCATTCATGC |
| 1975_3'R+Sac.1 | GGCGGAGCTCATTTTGCGGAACAGGATGAC |
| 1974_5'R.2(76) | ATTCCATTTGCTCAAGCGGGATCCCCTGTAATATCAGCCCAAT |
| 1976_3'F.2 | TGGGCTGATATTACAGGGGATCCCGCTTGAGCAAATGGAATTA |
| 1976_3'R.2 | ATGGTTTAGCTGCCCTCTCA |
| 1976_3'R+Sac.2 | GGCGGAGCTCATGGTTTAGCTGCCCTCTCA |
| 3153upF2 | AGCGCTTTGTTGTTTAGCAATG |
| 3153upR2+ | AACACCCGAGAAAATTCATCGATGGGGTTTGTTCCGGTTTATTTT |
| 3153dnF2+ | CGCACTAGCCAACAAGAGCTTCAAGGAAGGAATGGAACACTTACGC |
| 3153dnR2 | TGTAAACCACCGGAATGAATTG |
| 3260upF2 | TTACTTCACCCCAGACTCAGCA |
| 3260upR2+ | AACACCCGAGAAAATTCATCGATATTGCGATAATTGTTGGGTCGT |
| 3260dnF2+ | CGCACTAGCCAACAAGAGCTTCATGCAAAATCAATCATTCCAAGC |
| 3260dnR2 | ATTCCTGATATCCACCCACAGC |
| 3261dnF3+ | CGCACTAGCCAACAAGAGCTTCAAAGACACGCTTGAACAAGTATCTGA |
| 3261dnR3 | CTCCTGTCCCCCAACCAATAAT |
| 3261upF2 | AGCAAGCTCTGGAAGCATTACC |
| 3261upR2+ | AACACCCGAGAAAATTCATCGATATACAAGAATGGGCTCGCACTT |
| 3486upF2 | GCAAACGGGTCATGTTCTAGTG |
| 3486upR2+ | AACACCCGAGAAAATTCATCGATCCTGCTTGAGCAAACTGCGTA |
| 3486dnF2+ | CGCACTAGCCAACAAGAGCTTCATTGCCTGTGTTTTATTGCTCTCA |
| 3486dnR2 | AGAGGTCTGGTTTCAGCGTTTC |
| 3560upF2 | TTTTGGGAACTTGGACTCGTTT |
| 3560upR2+ | AACACCCGAGAAAATTCATCGATAATGCGACAATCAAAATCACTGG |
| 3560dnF2+ | CGCACTAGCCAACAAGAGCTTCACGACATTTAGCTGGGTTCGTTT |
| 3560dnR2 | ACCGCTTAGGTGACCAAACAAT |
| 4087_5'Fwd_1 | TGGCGGCAATACATAAAACA |
| 4087_5'Rev_1 | ATAGAAAAAGCAATAAATAACCAGGATCCGCTTAAGGAGATGTAAATGGGTTT |
| 4087_3'Fwd_1 | CATTTACATCTCCTTAAGCGGATCCTGGTTATTTATTGCTTTTTCTATATTCG |
| 4087_3'Rev_1 | TCACCTTCTCTTGTCGCTCA |
| 4087_5'Fwd+Pst_1 | GGCGCTGCAGTGGCGGCAATACATAAAACA |
| 4087_3'Rev+Sac_1 | GGCGGAGCTCTCACCTTCTCTTGTCGCTCA |
| RI1_5'_FW6 | CACCAACGTAGCGCACTTATACT |
| RI1_5'_RV6_Hom | TCACCCGGTTTGGGGTGTGAACCGCCCCCAACCAGTG |
| RI1_3'_FW6_Hom | CACTGGTTGGGGGCGGTTCACACCCCAAACCGGGTGA |
| RI1_3'_RV6 | GGTGGAATGGCTTTGTGGGTA |
| RI1_5'_FW6+Bam | GGCGGGATCCCACCAACGTAGCGCACTTATACT |
| RI1_5'_FW6+Hind | GGCGAAGCTTCACCAACGTAGCGCACTTATACT |
| RI1_3'_RV6+Sac | GGCGGAGCTCGGTGGAATGGCTTTGTGGGTA |
| RI2_5'_FW3 | TCAGGGCAGCTACTCAATTAC |
| RI2_5'_RV3_Hom | CTATAATAAACTTGTCTGCCTGACGCTTATTTCGACGTAAAATATCGATAATGTAG |
| RI2_3'_FW7 | CGTCAGGCAGACAAGTTTATTATAG |
| RI2_3'_RV7 | AAATCAGGTTCAGGCTTTGC |
| RI2_5'_FW3+Res | GGCGCTGCAGTCAGGGCAGCTACTCAATTAC |
| RI2_3'_RV7+Res | GGCGGAGCTCAAATCAGGTTCAGGCTTTGC |
