# Supplementary material for: Importance of Core Genome Functions for an Extreme Antibiotic Resistance Trait
Source: mBio. 2017 Dec 12;8(6):e01655-17. doi: 10.1128/mBio.01655-17 (PMC5727411; doi:10.1128/mBio.01655-17)
Supplement: TEXT S1 [file mbo006173636s1.docx]

**Supplemental Materials and Methods**

**Media.** The growth media used was LB (10 g tryptone, 5 g yeast extract, and 8 g NaCl per liter) solidified in LB agar with 15 g/l agar. For phosphate analysis, the media was MOPS minimal (1) with 15 mM sodium succinate and K_2_HPO_4_ at the levels indicated in Table 3, solidified with agarose.

**Plasmids.** Plasmid pSL15A was derived from pEX2-TetRA (from H. Kulasekara, unpublished), a derivative of pEXG2 (2) carrying Tn10 *tetR-tetA* and *sacB* in place of the *aacA1* gene. Plasmid pSL15A was constructed by deleting a 259 bp sequence between the *sacB* and *tetA* genes of pEX2-TetRA that carried an apparent high frequency recombination site. Plasmid pABcre was derived from pAT801-RA (3), and carries *cre* and a gene (*arr2*) conferring rifampicin resistance. Details of the plasmid constructions are available on request.

**Construction of targeted deletion mutations.** Targeted deletions of individual genes were created either by natural transformation of marked linear replacement constructs or by a suicide plasmid integration-excision method for introducing unmarked deletions.

For the natural transformation method, approximately 1,000 bp of genomic sequences flanking each intended deletion were amplified and joined by overlap extension PCR with the tetracycline resistance cassette from transposon T26 in place of the sequences to be deleted. The tetracycline resistance marker and flanking LoxP sites were amplified from plasmid pLG123 (4) using primers T26xLoxF2 and T26xLoxR2. Other primers used are listed in table S1, and primer sequences are reported in Table S2. The natural transformation procedure has been described (5). Transformants were selected on LB agar with 10 µg/ml tetracycline and the deletions were confirmed by PCR.

For the plasmid integration-excision method, approximately 1,000 bp of genome sequences flanking each intended deletion were amplified and joined by overlap extension PCR using primers which added restriction sites at both ends, and purified products were cloned into plasmid pSL15A. The primers used for individual deletions are reported in Tables S1 and S2. Plasmids were conjugated into *A. baumannii* from donor strain S17-1 λpir (6) or MFD*pir* (7). For conjugation, donor and recipient strains grown on solid media were suspended to visible turbidity in L broth (for donor strains, the media was supplemented with 15 µg/ml tetracycline and, for MFD*pir*, 0.3 mM DAP) and incubated for approximately 2 h at 37° with agitation. Following incubation, 0.75 ml of the donor cells and 1.0 ml of the recipient cells were washed twice with LB without supplements and pelleted, then resuspended together in 100 µl L broth. The mix was spotted onto 0.45-µm nitrocellulose filters (Merck Millipore, Ltd.) on pre-warmed LB agar, dried for approximately 15 min, and incubated at 37° for approximately 4 h. Cells were then washed from the filter with 1 ml L broth and plated for transconjugants on LB agar containing tetracycline (10 or 15 µg/ml) and 10 µg/ml chloramphenicol (S17-1 λpir donor). (MFD*pir* was counter-selected by the absence of DAP in the LB agar). Transconjugants were colony purified then streaked onto LB agar lacking NaCl and containing 6% sucrose to counter-select the plasmid *sacB* marker. Sucrose-resistant colonies were screened for the desired deletions by PCR, and confirmed to have lost the pSL15A vector based on their tetracycline sensitivity.

**Construction of strain AB5075ΔRI.** Strain AB5075ΔRI (MAB104) was constructed by precise sequential deletions of genome resistance islands RI2 and RI1. The suicide plasmid integration-excision method described in the previous section was used for both steps (primers used are reported in Tables S1 and S2). Single deletion mutants of RI1 and RI2 were also created (Dataset S2).

RI2 and its 5-bp target-site duplication (4) were replaced using a construct designed to restore the predicted ancestral gene, ABUW_4043/ABUW_4065 (Fig. 3). Sequencing of the restored ancestral gene in AB5075ΔRI2 revealed that three missense changes had been inadvertently introduced (corresponding to N30S, G169D and N170D).

RI1 represents an insertion in the *comM* gene with a 6-bp target-site duplication (8). Initial attempts at allelic replacement using a suicide plasmid construct designed to restore the wild-type *comM* gene resulted in a high frequency of merodiploids in which both copies of *comM* contained the RI1 insertion, suggesting that the island was actively transposing by a replicative mechanism into the newly-introduced wild-type copy of *comM*. We assumed that the transposition occurred by a Tn*7*-like mechanism and identified a potential recognition site, which we deleted from the incoming copy of *comM* (not shown). Using this deletion strain as a starting point, we were able to overcome the homogenotization problem and construct the desired RI1 deletion. Sequencing of the restored *comM* gene in AB5075ΔRI revealed two single nucleotide polymorphisms, corresponding to two synonymous changes in codons F60 and Y332.

**Construction of multiple and back-crossed mutants.** Double mutants were created from strains in the transposon mutant library by first excising the T26 tetracycline resistance determinant from a mutant, leaving a small, unmarked insertion, then transforming genomic DNA from a second mutant, with selection for tetracycline resistance. Triple mutants were made by repeating the process.

To excise the T26 tetracycline resistance marker from a mutant, plasmid pABcre was introduced by electroporation, with selection after outgrowth of transformants on LB agar containing 25 µg/ml rifampicin. Transformants were colony purified and patched to LB agar with and without 10 µg/ml tetracycline to screen for loss of tetracycline resistance. Tetracycline-sensitive strains were then cured of pABcre by growth to stationary phase in LB broth without rifampicin. Isolated colonies obtained by plating on LB agar were patched to LB agar with and without 25 µg/mL rifampicin to screen for loss of the plasmid.

Natural transformation with genomic DNA to introduce insertion mutations into new strain backgrounds employed approximately 200 ng DNA and followed established procedures (5). In addition to creating multiple mutants, the method was used to back-cross insertion alleles into AB5075 (Fig. 5).

**Growth of mutant pools in the presence of tobramycin.** For Tn-seq screens, 1-ml aliquots of the frozen mutant pool were thawed at room temperature for 30 min, incubated with agitation at 37°C for 30 min and then diluted in cold LB to a concentration of approximately 6.0 x 10^7^ cfu/ml. Pre-warmed 10-cm petri plates with L agar containing tobramycin or without drug were inoculated with 100 µl of this culture per plate (with multiple plates inoculated per drug level) and incubated at 37°C. The tobramycin levels used for AB5075 (MIC 32 µg/ml) were: no drug, 4.0, 6.0, 8.0 and 12.0 µg/ml. The levels used for AB5075ΔRI (MIC 2 µg/ml) were: no drug, 0.25, 0.375 and 0.625 µg/ml. Growth at each drug level was monitored by washing cells from individual plates into LB broth at different time points, measuring the OD_600_ and volume of the suspension, and calculating the predicted cell titer based on a standard curve. When growth per drug level reached the equivalent of ten doublings from the initial inoculum, the cells from five plates were harvested as above, mixed, and genomic DNA was isolated (Qiagen Tissue Kit; Qiagen, Inc.). Accurate viable cell titers of the inoculating culture and the final cell harvests were measured by serial dilution and plating.

**Tn-seq molecular processing.** Detailed Tn-seq protocols, including oligonucleotides used, are available upon request. Custom scripts (https://github.com/lg9/Tn-seq) were used to distinguish transposon from genomic sequences in raw Illumina sequences, map the reads to the AB5075 genome, tally reads per insertion site and read direction, and assign sites to annotated AB5075 genes. Summary read count and mapping data per sample are shown in Table S3. The sequence reads for each sample (after trimming the transposon-specific sequences) have been deposited in the Sequence Read Archive (SRA) (https://www.ncbi.nlm.nih.gov/sra) under accession numbers listed in Table S3.

**Tn-seq data analysis.** Read counts per sample were initially normalized to 10 million total reads and for local read density as described (9). The read counts were further normalized using a method similar to one used for RNA-Seq (10), as follows: (1) for each pool (the AB5075 pool and the AB5075ΔRI pool) a “pseudo reference sample” was created with read counts at each insertion location equal to the geometric mean of the read counts at that location across all samples for the pool, counting only insertion locations with reads in all of the samples; (2) a size factor was calculated for each sample as the median of the ratios of the sample reads to the pseudo reference sample reads, using the same set of locations used to define the pseudo reference sample; (3) the reads for each sample (at all locations) were normalized by dividing by that sample’s size factor.

Gene-level mutant fitness difference (fitness with tobramycin – fitness without tobramycin) was the primary criterion used to define genes negatively selected at each tobramycin level. Gene-level mutant fitness reflects the rate of expansion for hits within a gene relative to the entire pool (independent of total amount of pool growth), and was calculated based on a definition for fitness of individual insertions (11), but used total normalized reads from all insertion locations between the 5th and 90th percentile of the gene’s coding region: Fitness per gene = log ( d · W_post_ / W_pre_ ) / log ( d · (1 – W_post_) / (1 – W_pre_) ) where d = pool expansion (determined by titer measurements), W_post_ = frequency after growth, and W_pre_ = frequency before growth (frequency = total normalized reads within the gene / total normalized reads for the entire sample). Genes negatively selected at each tobramycin level were defined as those with a gene-level mutant fitness difference of less than -0.18. This value was chosen empirically as providing good discrimination of sensitive from unaffected mutants based on MIC assays of individual mutants. When the distribution of gene-level fitness differences for all genes was modeled by a normal distribution curve, the fitness difference cutoff value (-0.18) corresponded to a P value of less than 0.01 for all tobramycin levels analyzed. We further refined the list of negatively selected genes at each tobramycin level using two non-parametric statistical tests, which take advantage of the multiplicity of insertions within most genes to assign significance based on the consistency and degree of effect among the multiple insertions. The two tests were Wilcoxon rank sum tests that compared the set of all insertion locations within a gene (between the 5th and 90th percentile of the coding region) to the set of all insertion locations outside of the gene. For the first test, the metric under comparison was the absolute difference in normalized read counts per insertion location (reads after growth with drug – reads after growth without drug). By using read count differences as the metric of comparison, the influence of insertions with relatively low read counts in both samples (for which the signal-to-noise ratio tends to be high) was reduced. For the second test, the metric under comparison was the difference in normalized read count ranks per insertion location (read count rank after growth with drug – read count rank after growth without drug). By using read count rank as the metric of comparison, genes with overall low read counts but with multiple hits showing consistent differences (suggesting a real effect despite the high level of noise for such hits) were identified as significant. For each test, the calculated P values were adjusted for multiple comparisons using the Benjamini-Hochberg method. Genes were considered significantly negatively selected if they were non-essential (see below), displayed a gene-level mutant fitness difference of less than -0.18, and achieved an adjusted P value of less than 0.05 by either of the two Wilcoxon rank sum tests.

In addition to the genes implicated by the statistical analysis, ten genes were designated as significantly negatively selected based on manual inspection of their patterns of hits and reads (Dataset S1).

Genes significantly positively selected were defined as those showing significant negative selection described above, but requiring a gene-level fitness difference of greater than 0.18. The positively-selected genes listed in Dataset S1 are those which showed significant positive selection at two or more levels of tobramycin in either AB5075 or AB5075ΔRI (with exclusion of four genes based on manual examination of their patterns of hits and reads.)

**Gene essentiality.** Gene essentiality in each parent strain (AB5075 and AB5075ΔRI) was defined based on the Tn-seq data from the corresponding mutant pool before growth (the “pre” sample) as follows: (i) log_2_(reads per gene per gene Kbp) (l2rpkb) was calculated for each gene using the total normalized reads from all insertion locations between the 5th and 90th percentile of the gene’s coding region; (ii) a normal curve was fitted to the major peak of the distribution of l2rpkb values; (iii) genes with l2rpkb values below a cutoff corresponding to a P value of 0.001 for the normal curve were defined as essential.

**Core genes.** Core AB5075 genes were defined as those with homologs in ten or more of the following strain genomes, according to the Prokaryotic Genome Analysis Tool (http://tools.uwgenomics.org/cgi-bin/pgat_acinetobacter/genomelist.cgi): *A. baumannii* strains 1656-2, AB0057, AB307-0294, ACICU, ATCC 17978, ATCC 19606, AYE, D1279779, MDR-TJ, MDR-ZJ06, SDF, TCDC-AB0715, TYTH 1 and *A. baylyi* ADP1.

**Tobramycin sensitivity assays.** Individual strains were assayed for tobramycin sensitivity by minimal inhibitory concentration (MIC) assays of spot growth and efficiency of plating on LB agar containing antibiotic. For spot assays, 10 µl droplets containing ~5 X 10^3^-5 X 10^5^ viable cells in exponential phase growth were spotted on LB agar supplemented with different concentrations of tobramycin. The plates were incubated 16-18 h at 37°C, and MIC was scored visually as the lowest tobramycin concentration at which growth was inhibited. For efficiency of plating assays, dilutions of exponentially-growing cells (10^-1^ to 10^-7^) were spotted (8 µl per spot) onto LB agar containing tobramycin. Plates were incubated 15-16 h at 37°C and colonies counted. The MIC was defined as the lowest tobramycin concentration at which the plating efficiency was less than 5% of that on media without tobramycin. Fewer than 7% of the mutants initially screened corresponded to translucent colony variants (12). In our efficiency of plating assay, wild-type translucent variants displayed less than 2-fold decreased tobramycin MIC compared to opaque variants (not shown). Amikacin and Tobramycin MICs were determined by E-test strips following standard protocol on LB agar (bioMérieux).

**Phenotype microarrays.** Phenotype microarray analysis of AB5075, AB5075ΔRI, AB5075ΔRI1, AB5075ΔRI2 and seven transposon mutants (listed in Dataset S2) followed published protocols (13).

**References**

1. Neidhardt FC, Bloch PL, Smith DF. 1974. Culture medium for enterobacteria. J Bacteriol 119:736-47.

2. Rietsch A, Vallet-Gely I, Dove SL, Mekalanos JJ. 2005. ExsE, a secreted regulator of type III secretion genes in *Pseudomonas aeruginosa*. Proc Natl Acad Sci U S A 102:8006-11.

3. Heritier C, Poirel L, Lambert T, Nordmann P. 2005. Contribution of acquired carbapenem-hydrolyzing oxacillinases to carbapenem resistance in *Acinetobacter baumannii*. Antimicrob Agents Chemother 49:3198-202.

4. Gallagher LA, Ramage E, Weiss EJ, Radey M, Hayden HS, Held KG, Huse HK, Zurawski DV, Brittnacher MJ, Manoil C. 2015. Resources for Genetic and Genomic Analysis of Emerging Pathogen *Acinetobacter baumannii*. J Bacteriol 197:2027-35.

5. Jacobs AC, Thompson MG, Gebhardt M, Corey BW, Yildirim S, Shuman HA, Zurawski DV. 2014. Genetic Manipulation of *Acinetobacter baumannii*. Curr Protoc Microbiol 35:6G 2 1-11.

6. de Lorenzo V, Cases I, Herrero M, Timmis KN. 1993. Early and late responses of TOL promoters to pathway inducers: identification of postexponential promoters in *Pseudomonas putida* with lacZ-tet bicistronic reporters. J Bacteriol 175:6902-7.

7. Ferrieres L, Hemery G, Nham T, Guerout AM, Mazel D, Beloin C, Ghigo JM. 2010. Silent mischief: bacteriophage Mu insertions contaminate products of *Escherichia coli* random mutagenesis performed using suicidal transposon delivery plasmids mobilized by broad-host-range RP4 conjugative machinery. J Bacteriol 192:6418-27.

8. Adams MD, Goglin K, Molyneaux N, Hujer KM, Lavender H, Jamison JJ, MacDonald IJ, Martin KM, Russo T, Campagnari AA, Hujer AM, Bonomo RA, Gill SR. 2008. Comparative genome sequence analysis of multidrug-resistant *Acinetobacter baumannii*. J Bacteriol 190:8053-64.

9. Gallagher LA, Shendure J, Manoil C. 2011. Genome-scale identification of resistance functions in *Pseudomonas aeruginosa* using Tn-seq. MBio 2:e00315-10.

10. Anders S, Huber W. 2010. Differential expression analysis for sequence count data. Genome Biol 11:R106.

11. van Opijnen T, Bodi KL, Camilli A. 2009. Tn-seq: high-throughput parallel sequencing for fitness and genetic interaction studies in microorganisms. Nat Methods 6:767-72.

12. Tipton KA, Dimitrova D, Rather PN. 2015. Phase-Variable Control of Multiple Phenotypes in *Acinetobacter baumannii* Strain AB5075. J Bacteriol 197:2593-9.

13. Bochner BR, Gadzinski P, Panomitros E. 2001. Phenotype microarrays for high-throughput phenotypic testing and assay of gene function. Genome Res 11:1246-55.
