# Supplementary material for: Importance of Core Genome Functions for an Extreme Antibiotic Resistance Trait
Source: mBio. 2017 Dec 12;8(6):e01655-17. doi: 10.1128/mBio.01655-17 (PMC5727411; doi:10.1128/mBio.01655-17)
Supplement: TABLE S3 [file mbo006173636st3.docx]

**Table S3. Tn-seq run summary information.**

| Run name | Pool | Tobramycin (µg/ml) | Expansion* | Total reads† | Mapped reads | Positions‡ | SRA Accession |
| --- | --- | --- | --- | --- | --- | --- | --- |
| Pre3^§^ | AB5075 | (pre-growth) | NA | 12,805,746 | 10,898,795 | 453,601 | SRR5893020 |
| Pre13^§^ | AB5075 | (pre-growth) | NA | 4,050,501 | 3,016,033 | 357,856 | SRR5893017 |
| Pre11^§^ | AB5075 | (pre-growth) | NA | 6,366,158 | 4,819,385 | 248,532 | SRR5893026 |
| Pre17^§^ | AB5075 | (pre-growth) | NA | 7,912,131 | 5,250,115 | 290,639 | SRR5893028 |
| TM0_1^¶^ | AB5075 | 0 | 2048.0 | 15,239,364 | 8,068,930 | 247,864 | SRR5893027 |
| TM0_2^¶^ | AB5075 | 0 | 1573.8 | 4,342,708 | 3,479,398 | 391,818 | SRR5893025 |
| TM4 | AB5075 | 4.0 | 1871.5 | 5,192,397 | 4,167,834 | 377,570 | SRR5893022 |
| TM6 | AB5075 | 6.0 | 2452.4 | 4,536,117 | 3,651,085 | 373,323 | SRR5893023 |
| TM8 | AB5075 | 8.0 | 2164.8 | 4,743,913 | 3,826,992 | 359,032 | SRR5893015 |
| TM12 | AB5075 | 12.0 | 2836.7 | 29,520,289 | 27,443,608 | 220,623 | SRR5893016 |
| dRIpre | AB5075ΔRI | (pre-growth) | NA | 3,364,306 | 3,159,927 | 124,004 | SRR5893014 |
| dRITM0 | AB5075ΔRI | 0 | 3061.5 | 3,279,007 | 3,081,683 | 119,227 | SRR5893021 |
| dRITM.2 | AB5075ΔRI | 0.25 | 3104.2 | 3,913,154 | 3,686,082 | 116,480 | SRR5893018 |
| dRITM.3 | AB5075ΔRI | 0.375 | 2076.6 | 3,990,818 | 3,762,487 | 111,787 | SRR5893019 |
| dRITM.6 | AB5075ΔRI | 0.625 | 1144.1 | 3,568,074 | 3,355,139 | 91,774 | SRR5893024 |

* The total expansion of the pool during the course of selective growth, determined from titer measurements before and after growth. NA, not applicable.

† Reads passing Illumina quality filtering and containing the expected sequence (with at most one mismatched base) for the transposon portion of the read.

‡ To account for possible polymerase slippage during Illumina sequencing (L. A. Gallagher, J. Shendure and C. Manoil, mBio 2(3):e00315-10, 2011, doi: 10.1128/mBio.00315-10), reads mapping to the same strand and to positions within two bp of one another were all assigned to the position with the greater read counts if the raw counts at that position were at least twenty times the raw counts at the other position.

§ The averages of the normalized reads per position for the four technical replicates of the AB5075 starting pool (Pre3, Pre13, Pre11 and pre17) were used as the pre-growth counts for fitness calculations following selective growth.

¶ Sample TM0_2 served as the no-drug comparison sample for samples TM4, TM6 and TM8 (all four selections were done together). Sample TM0_1 served as the no-drug comparison sample for sample TM12 (both selections were done separately from the other four). Normalized read counts were highly correlated between samples TM0_1 and TM0_2, with the exception of counts for insertions in plasmid p1, which were slightly underrepresented in sample TM0_1 relative to sample TM0_2. Plasmid p1 read counts for samples TM4, TM6 and TM8 were highly similar to those for sample TM0_2, while plasmid p1 read counts for sample TM12 were more similar to those for sample TM0_1.

SRA, Sequence Read Archive (<https://www.ncbi.nlm.nih.gov/sra>).
